# Labial Fusion Recurrence in a Prepubertal Girl: A Case Report and Review of Literature

**DOI:** 10.7759/cureus.26059

**Published:** 2022-06-18

**Authors:** Ipsita Mohapatra, Subha R Samantaray

**Affiliations:** 1 Obstetrics and Gynecology, All India Institute of Medical Sciences, Kalyani, Kalyani, IND

**Keywords:** prepubertal age, dysuria, conservative management, recurrence, labial fusion

## Abstract

Labial fusion is the fusion of labia minora or majora and results in partial or complete fusion of the vaginal orifice. The condition is commonly seen in prepubertal girls and post-menopausal women, but it can sometimes be seen in reproductive age group women also.

We present here a case of a three-year-old girl who presented with recurrence of labial fusion and dysuria. The patient had a similar labial fusion three months before, which was treated with manual separation followed by estrogen cream application. The recurrence of labial fusion was managed conservatively. This case report highlights the possibility of recurrence of labial fusion in prepubertal girls till they attain puberty, as well as its conservative management.

## Introduction

Labial fusion, also known as labial adhesion or labial agglutination, or labial synechiae is the fusion of labia minora or majora, most commonly near the clitoris. It can result in the development of thin fibrotic tissue and results in partial or complete fusion of the vaginal orifice [1].

It is often an acquired condition, can also occur as a result of congenital anomaly, and sometimes co-occur with other genitourinary abnormalities. Labial fusion is a common occurrence in prepubertal girls with an incidence of about 0.6%-5%, with the peak incidence of around 3% occurring in the second year of life [2,3]. The second common occurrence is among post-menopausal women due to the low estrogen levels during this age.

We present here a case of labial fusion in a three-year-old girl who presented with recurrence of the condition. She was treated with conservative management.

## Case presentation

A three-year-old girl was brought to the gynecology outpatient department by her mother with the chief complaint of labial adhesion and difficulty in urination. The mother was very anxious as there was a similar episode of vaginal occlusion with urinary retention three months back. The child gave a history of pain during micturition. There was also the history of wetting of the undergarments for about a week. The girl frequently had urinary symptoms, recurring urinary tract infections, and white leucorrhea. There was no history of any trauma, sexual abuse, or pinworm infestation.

**Figure 1:**
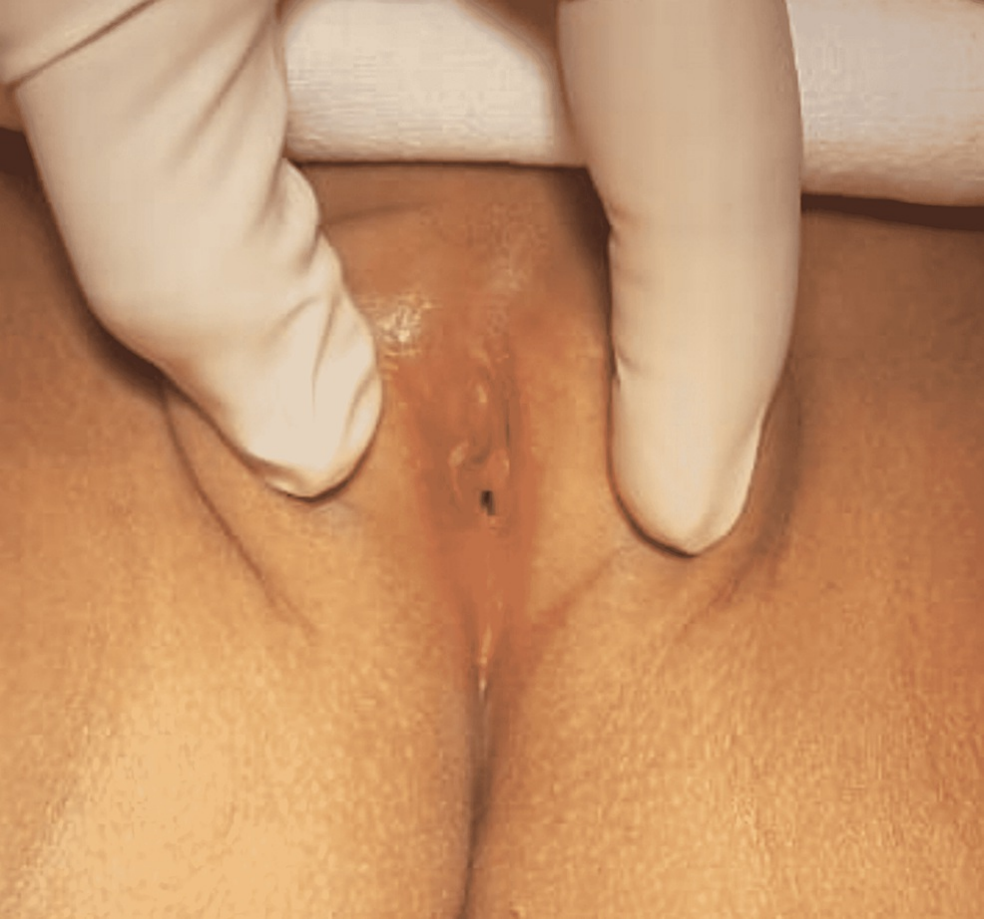
Near-total labial fusion with only a 2-mm opening at the center

The child had normal height and weight for her age and had achieved all developmental milestones. On abdominal examination, bladder distension was elicited. Examination of external genitalia revealed a complete fusion of labia minora with a very small opening (Figure 1). The mother had also noticed complete fusion of labia with complete stoppage of urination for three months for which she had consulted a pediatrician. At that time, she was advised to use estrogen-based cream and a pediatric Foley catheter was given for a period of one week. The condition had improved over two weeks. But now again there was near closure of the introitus of the vagina along with dysuria.

On trying to manually separate the labia, there was pain and the girl did not allow further manipulation.

Urine routine microscopic and culture revealed minimal growth of Escherichia coli, which was treated with oral antibiotics. Stool examination ruled out any infestation. As labial fusion is a clinical diagnosis, no other laboratory or imaging investigations were ordered. We advised local application of topical steroid (0.5% Betamethasone) ointment twice daily for 15 days, and on follow-up of the child, there was an improvement in the condition.

The parents were counseled about the possibility of recurrence of the condition and hence suggested regular follow-up. They were advised to continue steroid application for 15 more days and to maintain the local hygiene of the child.

## Discussion

Labial fusion is a condition mostly seen in prepubertal and post-menopausal women, which is due to low estrogen levels during these phases of life [2,3]. It is rare in the reproductive age group.

The pathology behind the development of labial fusion is little known. The most accepted theory is chronic inflammation and irritation and constant friction during mobility, which leads to superficial skin epithelium denudation and slow fusion of the labia in a low-estrogen environment [4]. Though the exact cause in the reproductive age group is unknown, it is suspected that labial adhesions may be caused by irritation and inflammation of external genitalia. These irritants can be urine, feces, soaps with artificial perfumes, poor hygiene leading to inflammatory conditions such as vulvitis, pinworm infestation, atopic dermatitis, any trauma due to delivery, or sexual abuse or conditions that may lead to hypoestrogenism [4]. Some cases of labial agglutination have been reported in the post-partum period also, which is believed to be due to breastfeeding compounded with vaginal trauma during childbirth [1]. During breastfeeding, due to high prolactin levels, ovulation is suppressed and estrogen levels are decreased.

Most cases of labial fusion are asymptomatic and are incidentally found out by the caretaker. Symptomatic cases may present with inflammation of the affected area, post-void dripping of urine, dysuria, hematuria, and rarely infertility [5]. Retention of urine may lead to urinary tract infections in severe cases. The inflammation may be caused due to infections or poor hygiene.

This condition usually resolves spontaneously at puberty in up to 80% of girls as the effect of estrogen changes the cells that line the genitals [6]. Hence, proper counseling and reassurance are the first lines of management. Symptomatic cases of labial fusion in young girls need medical management. Conjugated estrogen cream or estradiol vaginal cream (0.01%) can be applied to the adhesions one to two times daily for three to four weeks or until the adhesion resolves [7]. Mupirocin ointment has also been used for the improvement of the condition. An alternative form of medical management is the topical application of 0.05% betamethasone cream twice daily for four to six weeks [8]. A retrospective study that compared medical treatment with estrogen cream alone, betamethasone cream alone, and a combination of both for two to four weeks concluded that there were no significant differences among the study groups [9]. In developing countries where patients do not come for follow-up, steroid or estrogen cream cannot be given due to the risk of potential side effects, which can be offered either through manual or surgical separation of the fused labia.

Surgical separation is needed in cases of thick fusion that are not separable or are difficult for manual separation. Some severe cases (around 5-10%) that are non-responsive to medical treatment and recurrent cases also require surgical adhesiolysis [10,11]. But irrespective of the mode of treatment, the recurrence rate is found to be around 40% [12]. Surgical separation of the labial fusion is found to have a less recurrence rate than manual separation [6,13]. An uncommon cause of recurrence, such as congenital adrenal hyperplasia, may be looked at if there are multiple recurrences [14].

According to the response to the treatment, labial fusion has been divided into four categories: type 1 includes cases that completely resolve with topical steroid treatment in two weeks, type II includes cases that have a complete response to topical steroid treatment for an average of three weeks, and types III and IV include cases that are completely unresponsive to topical steroid treatment [15].

Prolonged treatment with topical estrogen or betamethasone may cause side effects such as hyperpigmentation of the labia and breast development. There can be a rare possibility of vaginal bleeding and precocious puberty also [16]. Therefore, the use of estrogen and or steroids in prepubertal girls should be guarded and for a short duration only.

The parents of a small girl with labial fusion may be worried about the possibility of an absent vagina. The conditions that may be considered as the differential diagnosis are imperforate hymen, hymenal skin tags, urethral prolapse, and vaginal atresia [1]. Parents have to be counseled properly about this benign condition, as well as its cause, treatment, and possible recurrence.

## Conclusions

Labial fusion in prepubertal girls though not uncommon is a matter of concern as neglected cases may report severe symptoms of frequent urinary tract infection. Proper assessment of the condition, reassurance of the parents, and judicious use of estrogen or steroids for treatment are mandatory. Recurrence of labial fusion can occur even after treatment and may be again managed by conservative treatment.
